# Supplementary figures and images for: A MSTNDel73C mutation with FGF5 knockout sheep by CRISPR/Cas9 promotes skeletal muscle myofiber hyperplasia
Source: eLife. 2024 Oct 4;12:RP86827. doi: 10.7554/eLife.86827 (PMC11452178; doi:10.7554/eLife.86827)

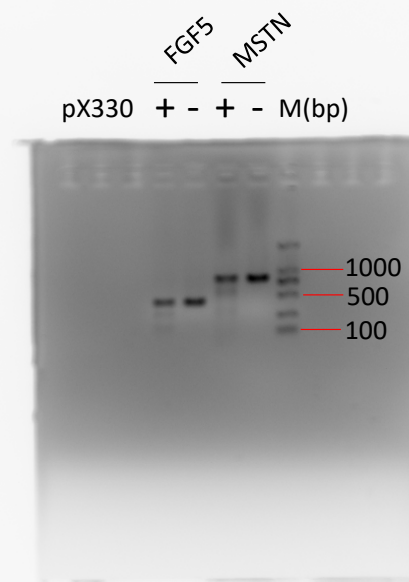

Supplement: Figure 1—source data 1. [file elife-86827-fig1-data1.zip › Figure 1-Source data 1 Uncropped and labeled gels for Figure 1.pdf]

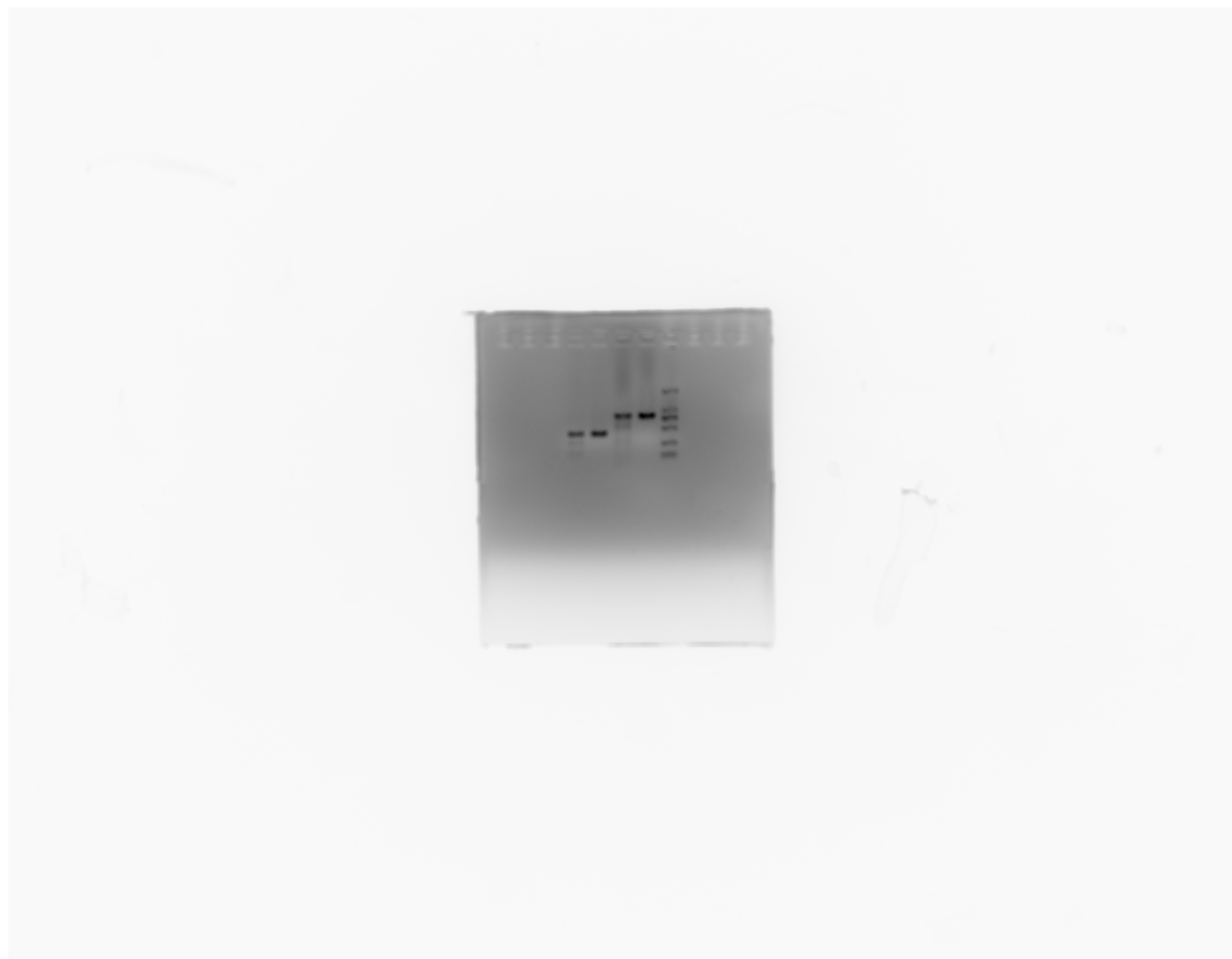

Supplement: Figure 1—source data 2. [file elife-86827-fig1-data2.zip › Figure 1-Source data 1 Raw unedited gels for Figure 1.pdf]

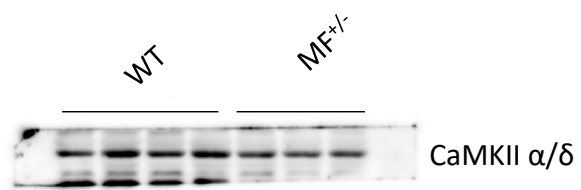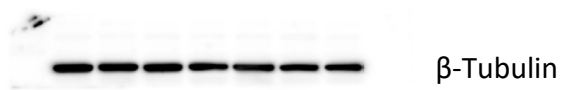

Supplement: Figure 2—figure supplement 2—source data 1. [file elife-86827-fig2-figsupp2-data1.zip › Figure 2-figure supplement 2-Source data 1 Uncropped and labeled blots for Figure 2-figure supplement 2.pdf]

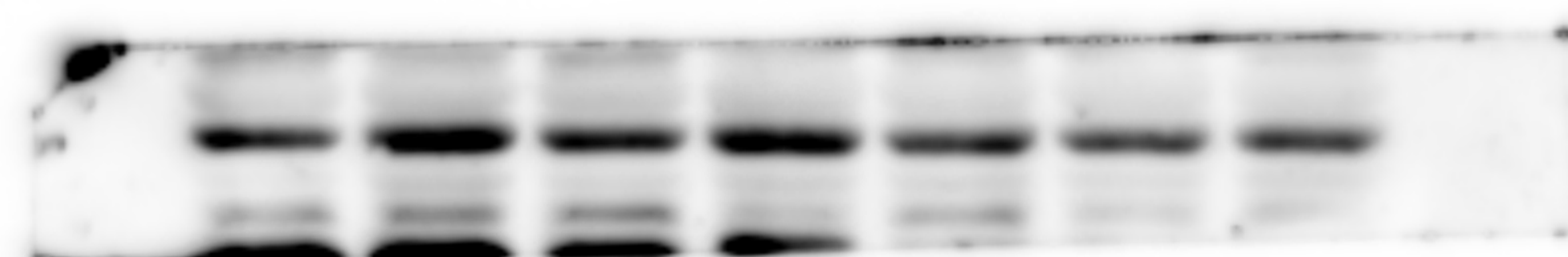

Supplement: Figure 2—figure supplement 2—source data 2. [file elife-86827-fig2-figsupp2-data2.zip › Figure 2-figure supplement 2-Source data 1 Raw unedited blots for Figure 2-figure supplement 2.pdf]

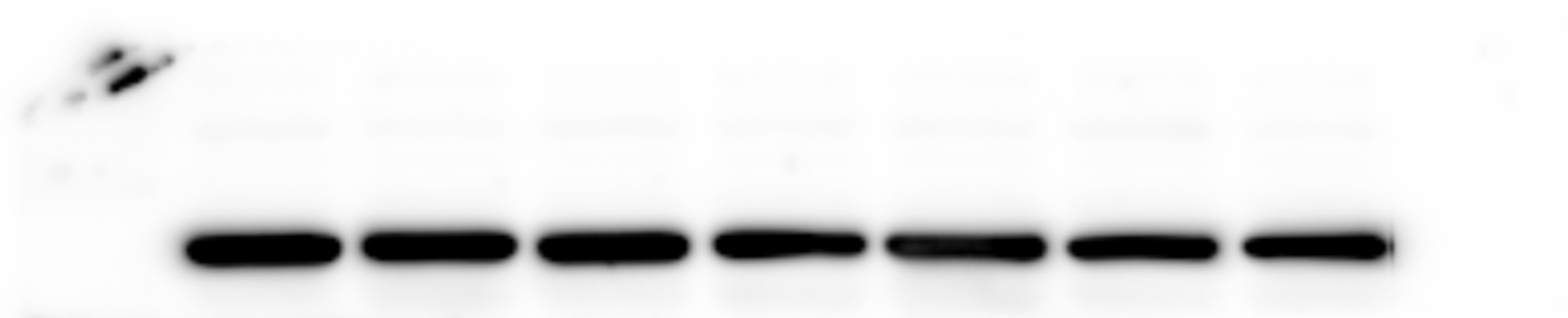

Supplement: Figure 2—figure supplement 2—source data 2. [file elife-86827-fig2-figsupp2-data2.zip › Figure 2-figure supplement 2-Source data 2 Raw unedited blots for Figure 2-figure supplement 2.pdf]

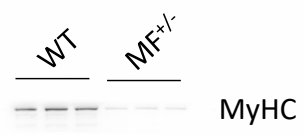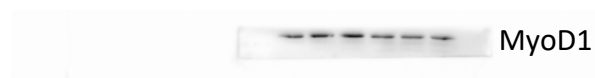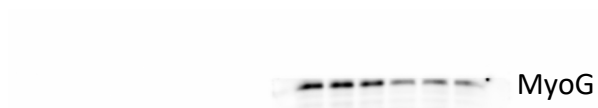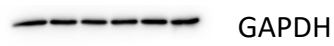

Supplement: Figure 3—source data 1. [file elife-86827-fig3-data1.zip › Figure 3-Source data 1 Uncropped and labeled blots for Figure 3.pdf]

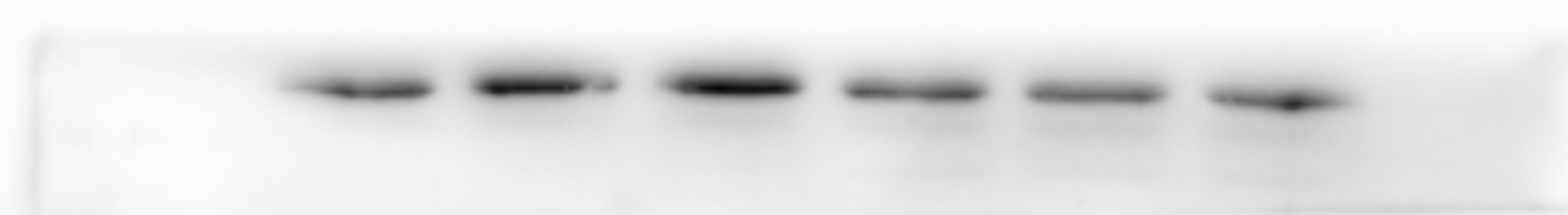

Supplement: Figure 3—source data 2. [file elife-86827-fig3-data2.zip › Figure 3-Source data 2 Raw unedited blots for Figure 3.pdf]

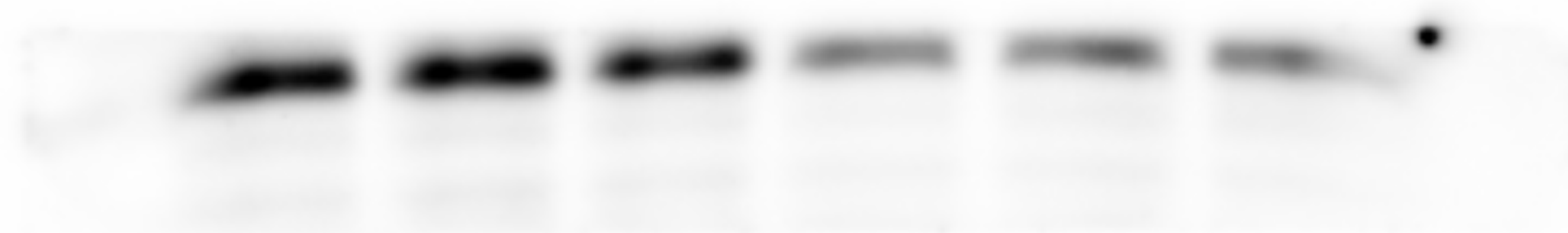

Supplement: Figure 3—source data 2. [file elife-86827-fig3-data2.zip › Figure 3-Source data 3 Raw unedited blots for Figure 3.pdf]

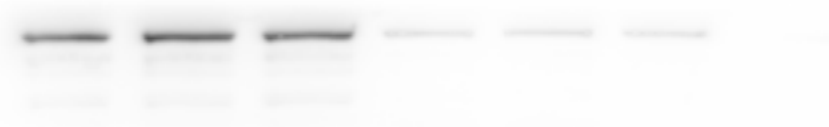

Supplement: Figure 3—source data 2. [file elife-86827-fig3-data2.zip › Figure 3-Source data 1 Raw unedited blots for Figure 3.pdf]

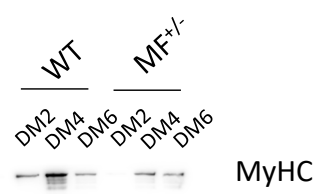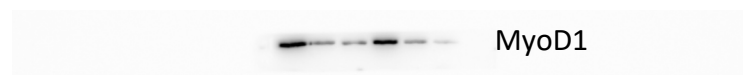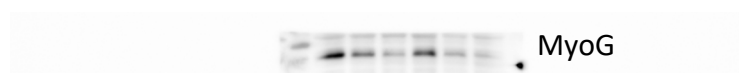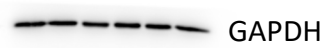

Supplement: Figure 3—figure supplement 1—source data 1. [file elife-86827-fig3-figsupp1-data1.zip › Figure 3-figure supplement 1-Source data 1 Uncropped and labeled blots for Figure 3-figure supplement 1.pdf]

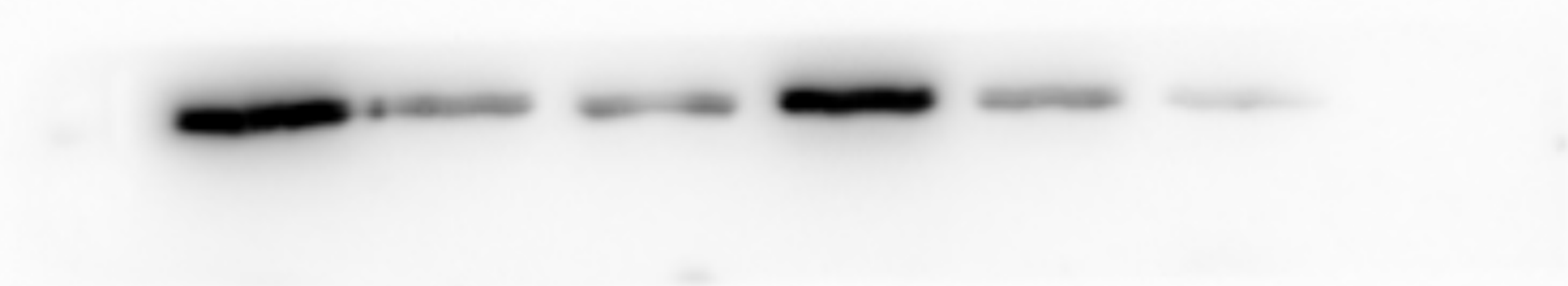

Supplement: Figure 3—figure supplement 1—source data 2. [file elife-86827-fig3-figsupp1-data2.zip › Figure 3-figure supplement 1-Source data 2 Raw unedited blots for Figure 3-figure supplement 1.pdf]

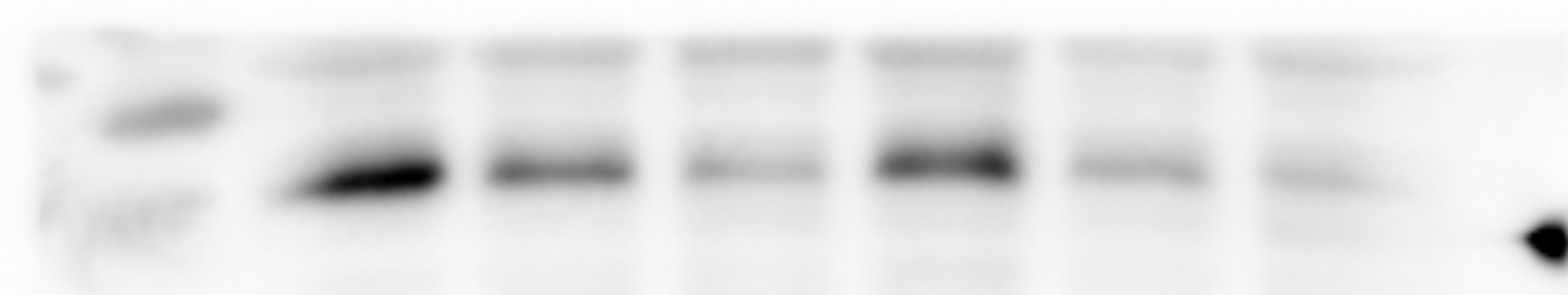

Supplement: Figure 3—figure supplement 1—source data 2. [file elife-86827-fig3-figsupp1-data2.zip › Figure 3-figure supplement 1-Source data 3 Raw unedited blots for Figure 3-figure supplement 1.pdf]

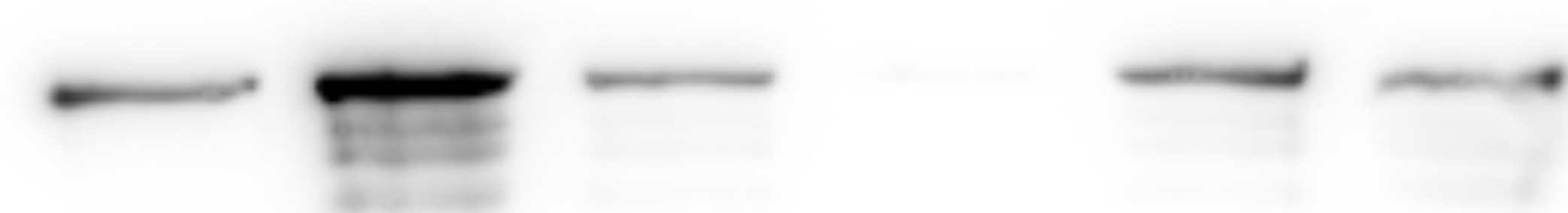

Supplement: Figure 3—figure supplement 1—source data 2. [file elife-86827-fig3-figsupp1-data2.zip › Figure 3-figure supplement 1-Source data 1 Raw unedited blots for Figure 3-figure supplement 1.pdf]

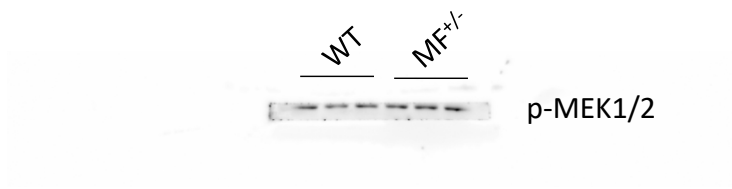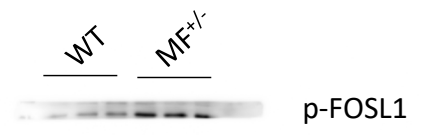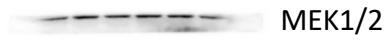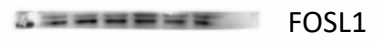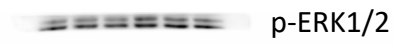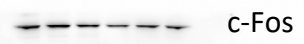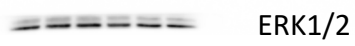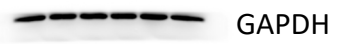

Supplement: Figure 4—source data 1. [file elife-86827-fig4-data1.zip › Figure 4-Source data 1 Uncropped and labeled blots for Figure 4.pdf]

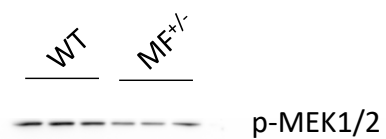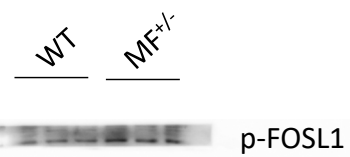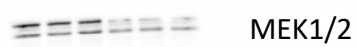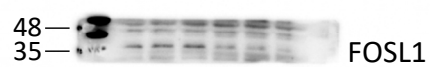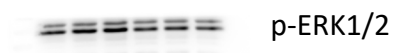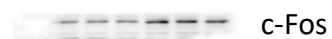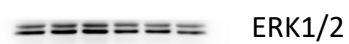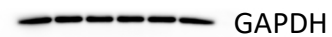

Supplement: Figure 4—source data 1. [file elife-86827-fig4-data1.zip › Figure 4-Source data 2 Uncropped and labeled blots for Figure 4.pdf]

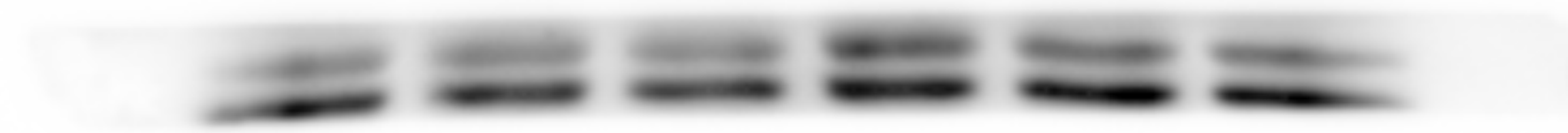

Supplement: Figure 4—source data 2. [file elife-86827-fig4-data2.zip › Figure 4-Source data 3 Raw unedited blots for Figure 4.pdf]

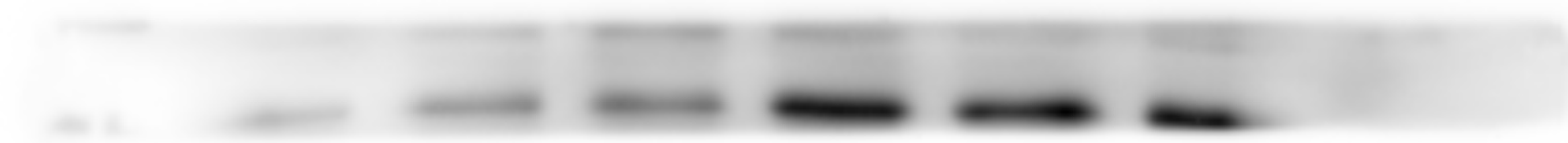

Supplement: Figure 4—source data 2. [file elife-86827-fig4-data2.zip › Figure 4-Source data 5 Raw unedited blots for Figure 4.pdf]

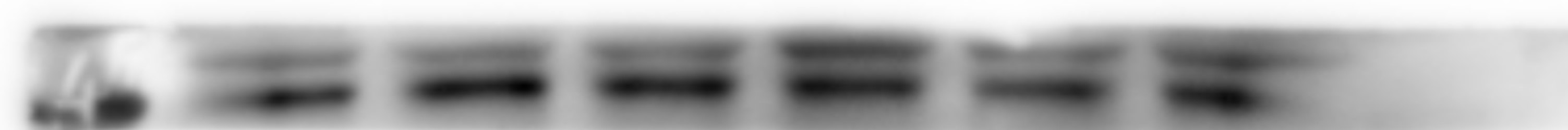

Supplement: Figure 4—source data 2. [file elife-86827-fig4-data2.zip › Figure 4-Source data 6 Raw unedited blots for Figure 4.pdf]

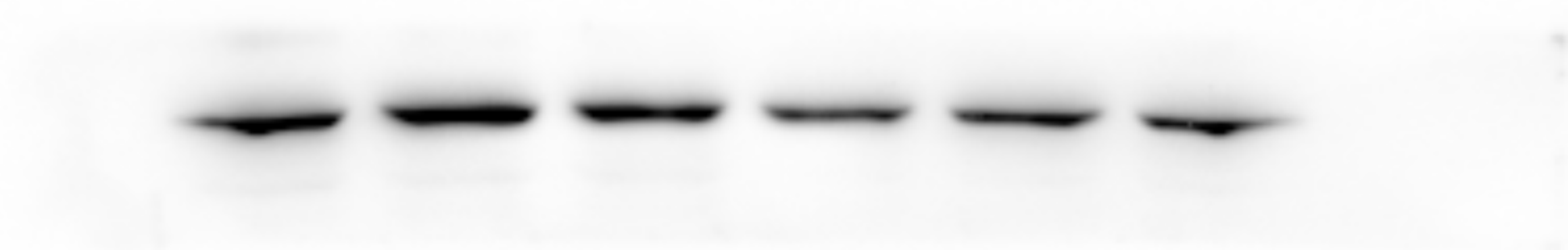

Supplement: Figure 4—source data 2. [file elife-86827-fig4-data2.zip › Figure 4-Source data 7 Raw unedited blots for Figure 4.pdf]

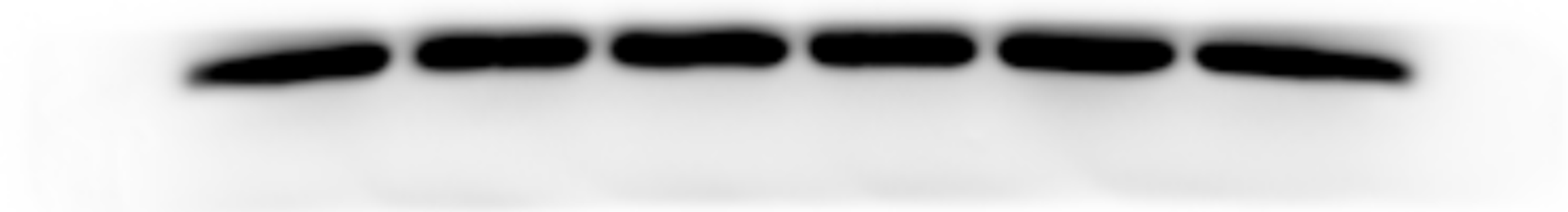

Supplement: Figure 4—source data 2. [file elife-86827-fig4-data2.zip › Figure 4-Source data 8 Raw unedited blots for Figure 4.pdf]

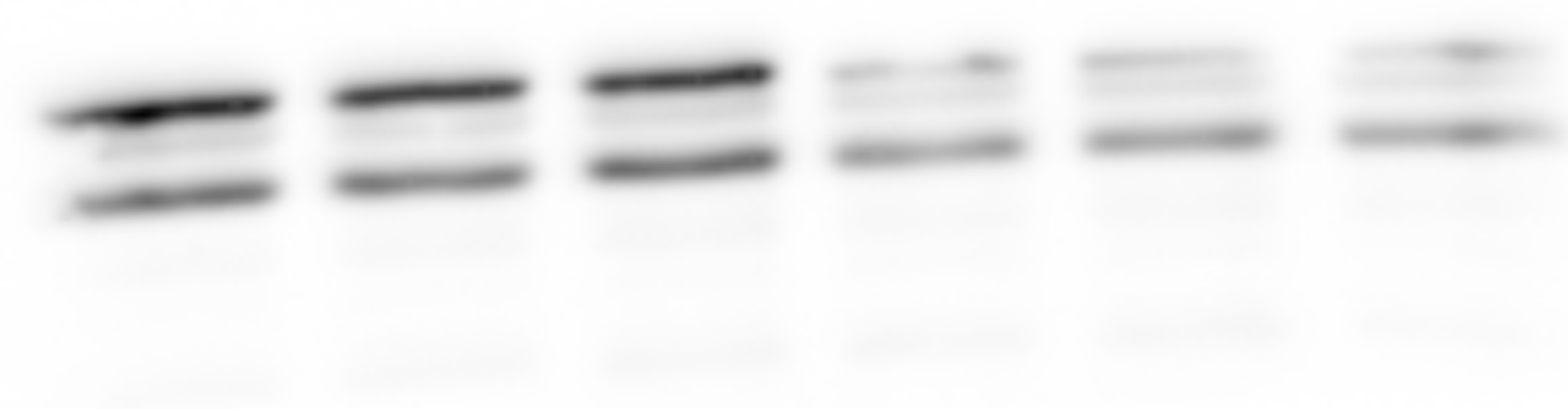

Supplement: Figure 4—source data 2. [file elife-86827-fig4-data2.zip › Figure 4-Source data 10 Raw unedited blots for Figure 4.pdf]

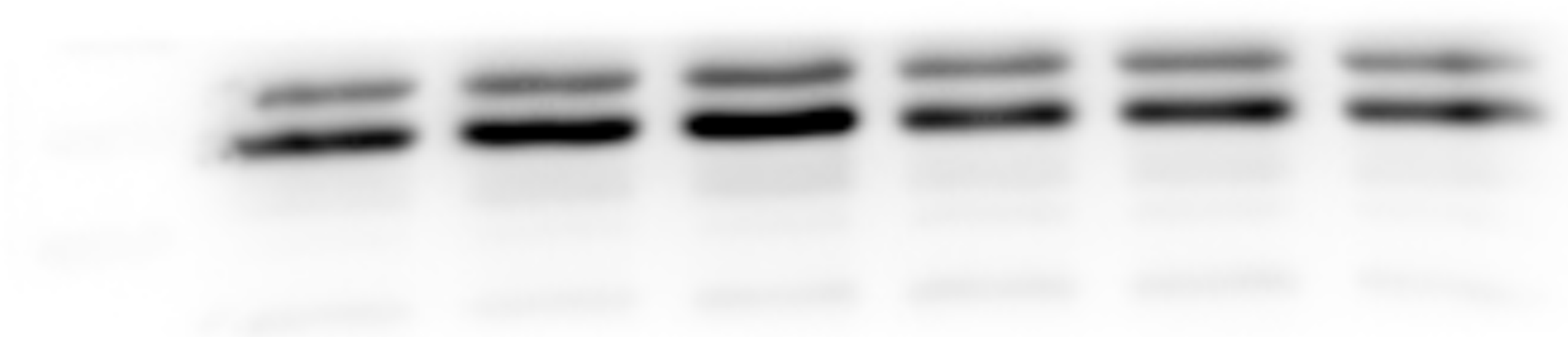

Supplement: Figure 4—source data 2. [file elife-86827-fig4-data2.zip › Figure 4-Source data 11 Raw unedited blots for Figure 4.pdf]

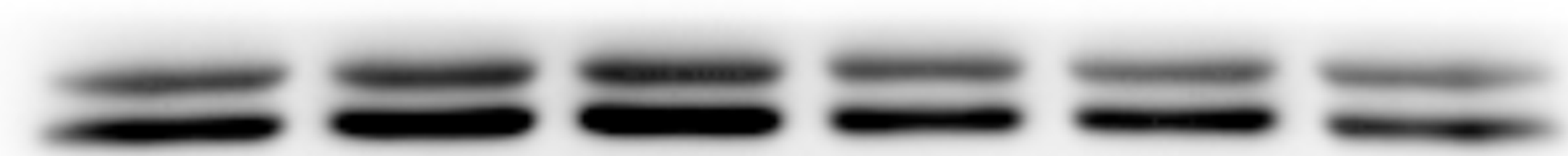

Supplement: Figure 4—source data 2. [file elife-86827-fig4-data2.zip › Figure 4-Source data 12 Raw unedited blots for Figure 4.pdf]

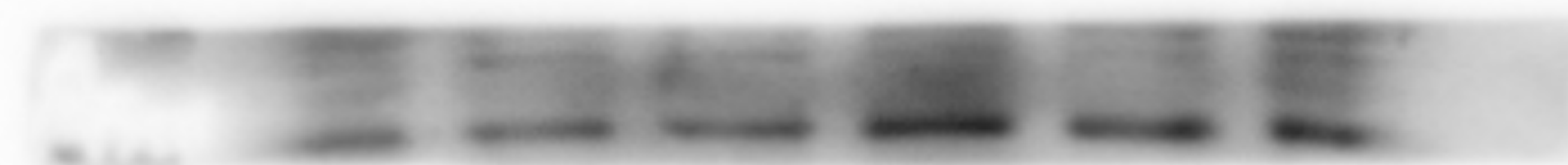

Supplement: Figure 4—source data 2. [file elife-86827-fig4-data2.zip › Figure 4-Source data 13 Raw unedited blots for Figure 4.pdf]

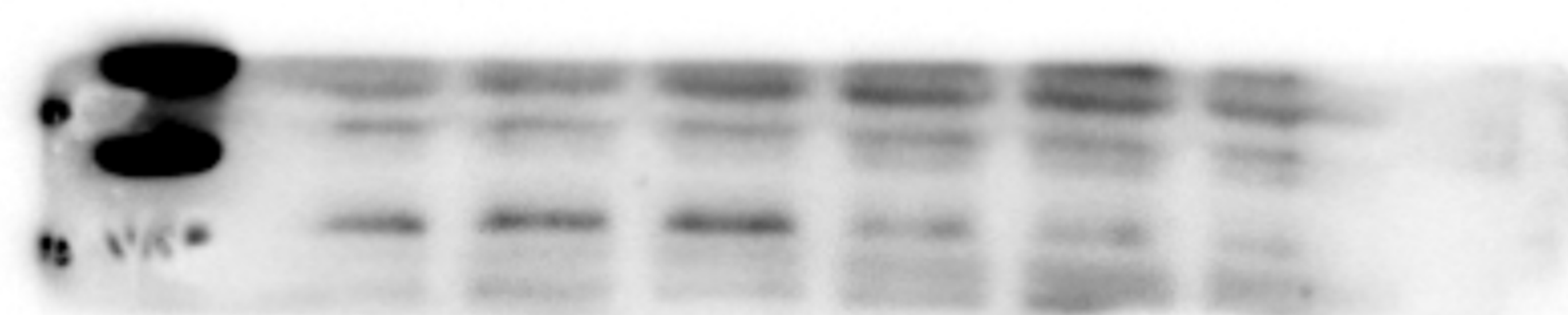

Supplement: Figure 4—source data 2. [file elife-86827-fig4-data2.zip › Figure 4-Source data 14 Raw unedited blots for Figure 4.pdf]

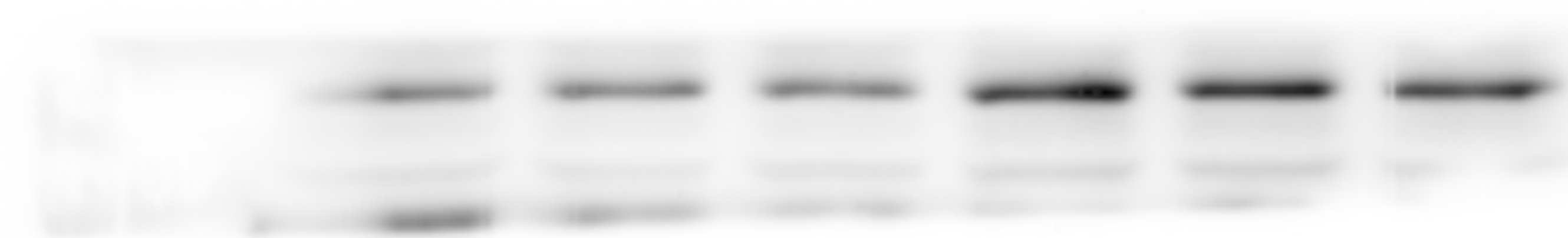

Supplement: Figure 4—source data 2. [file elife-86827-fig4-data2.zip › Figure 4-Source data 15 Raw unedited blots for Figure 4.pdf]

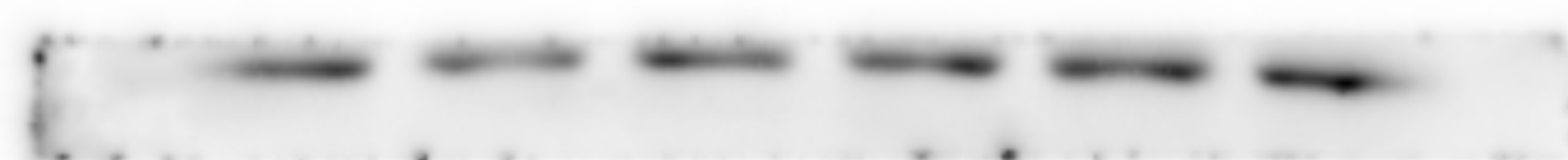

Supplement: Figure 4—source data 2. [file elife-86827-fig4-data2.zip › Figure 4-Source data 1 Raw unedited blots for Figure 4.pdf]

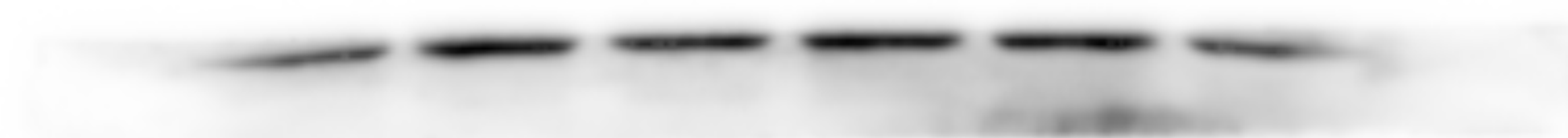

Supplement: Figure 4—source data 2. [file elife-86827-fig4-data2.zip › Figure 4-Source data 2 Raw unedited blots for Figure 4.pdf]

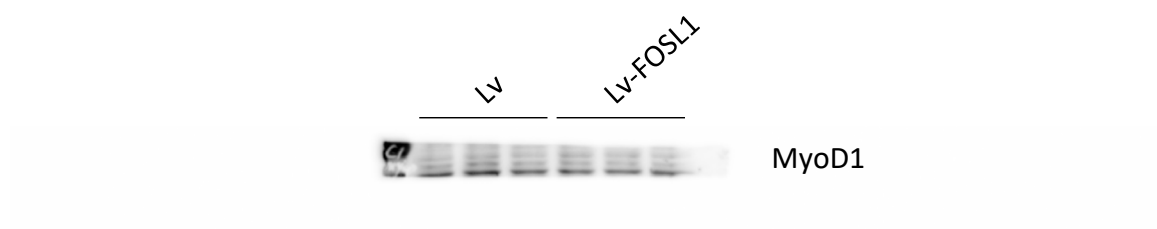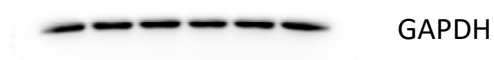

Supplement: Figure 5—source data 1. [file elife-86827-fig5-data1.zip › Figure 5-Source data 1 Uncropped and labeled blots for Figure 5.pdf]

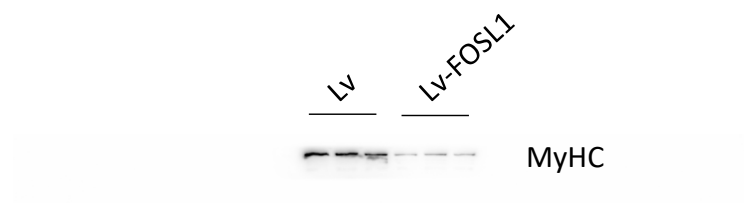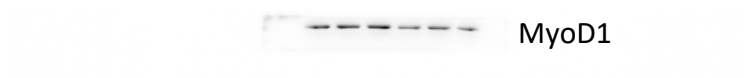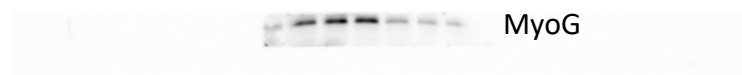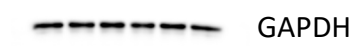

Supplement: Figure 5—source data 1. [file elife-86827-fig5-data1.zip › Figure 5-Source data 2 Uncropped and labeled blots for Figure 5.pdf]

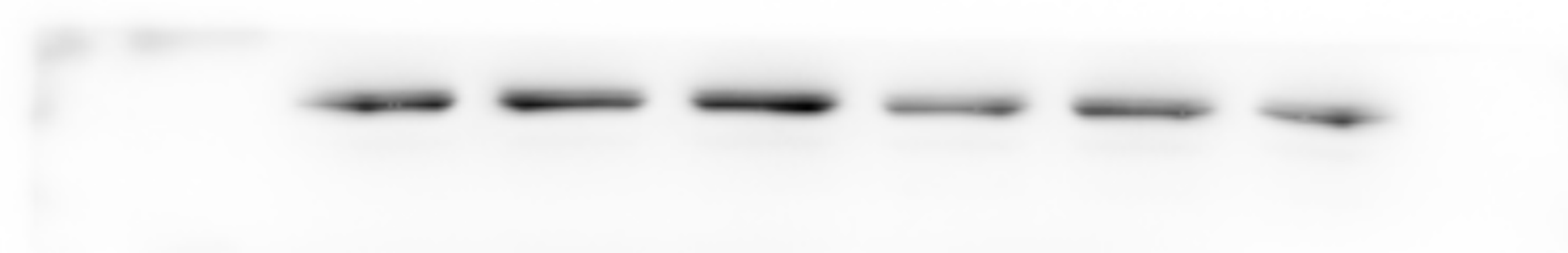

Supplement: Figure 5—source data 2. [file elife-86827-fig5-data2.zip › Figure 5-Source data 4 Raw unedited blots for Figure 5.pdf]

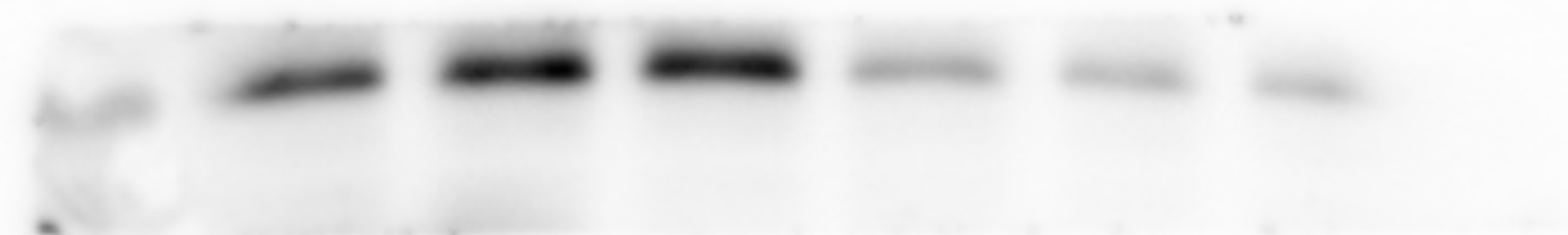

Supplement: Figure 5—source data 2. [file elife-86827-fig5-data2.zip › Figure 5-Source data 5 Raw unedited blots for Figure 5.pdf]

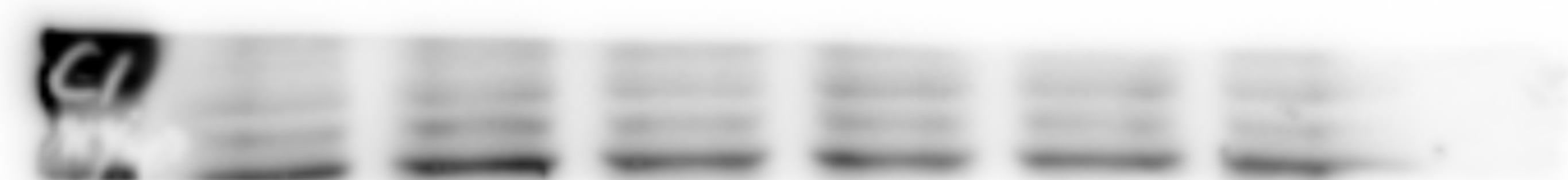

Supplement: Figure 5—source data 2. [file elife-86827-fig5-data2.zip › Figure 5-Source data 1 Raw unedited blots for Figure 5.pdf]

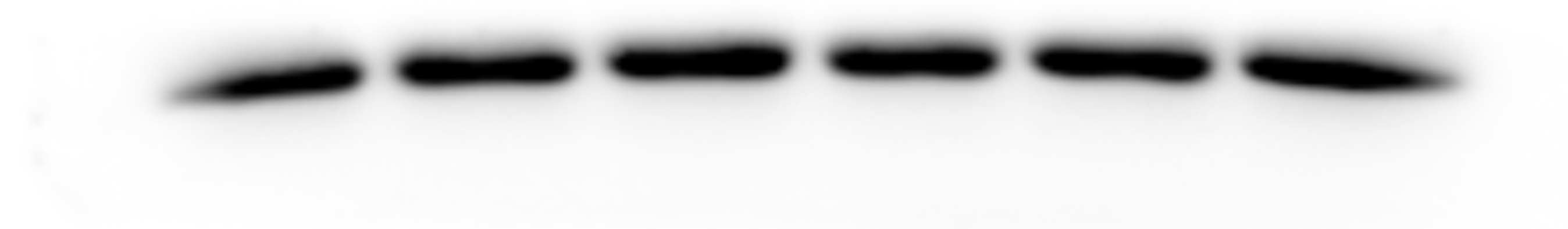

Supplement: Figure 5—source data 2. [file elife-86827-fig5-data2.zip › Figure 5-Source data 2 Raw unedited blots for Figure 5.pdf]

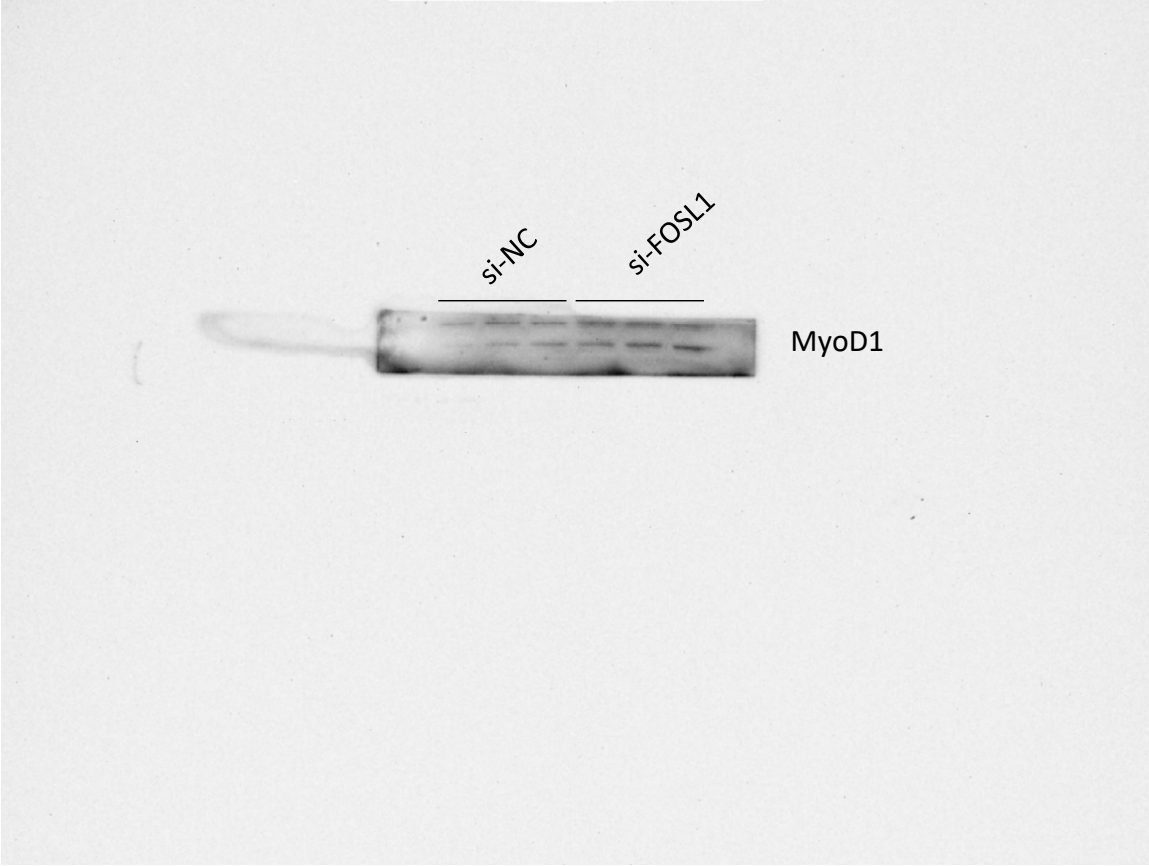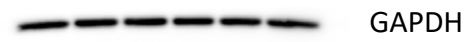

Supplement: Figure 6—source data 1. [file elife-86827-fig6-data1.zip › Figure 6-Source data 1 Uncropped and labeled blots for Figure 6.pdf]

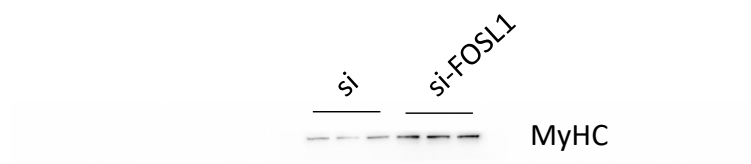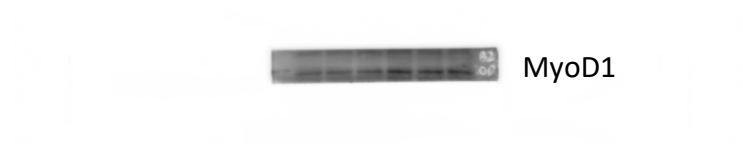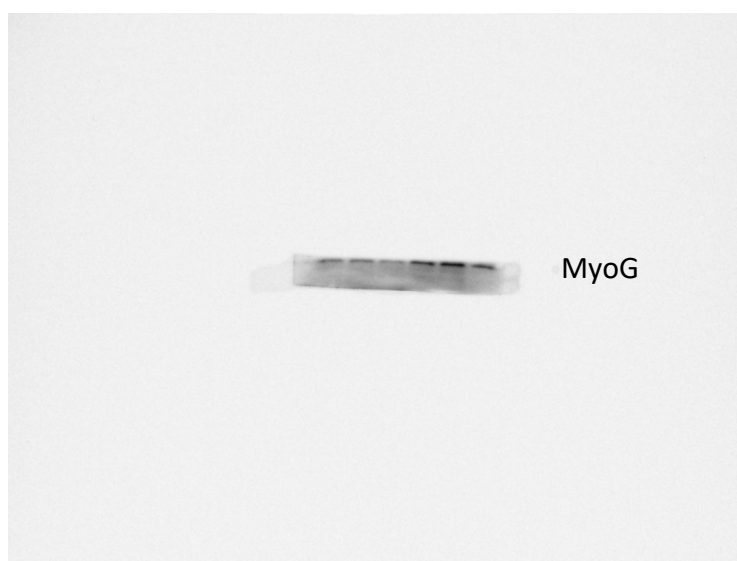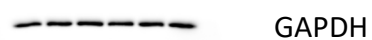

Supplement: Figure 6—source data 1. [file elife-86827-fig6-data1.zip › Figure 6-Source data 2 Uncropped and labeled blots for Figure 6.pdf]

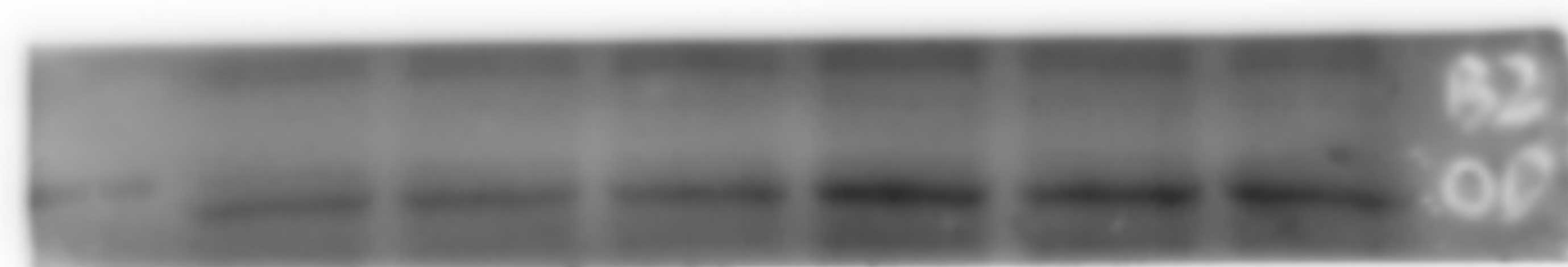

Supplement: Figure 6—source data 2. [file elife-86827-fig6-data2.zip › Figure 6-Source data 4 Raw unedited blots for Figure 6.pdf]

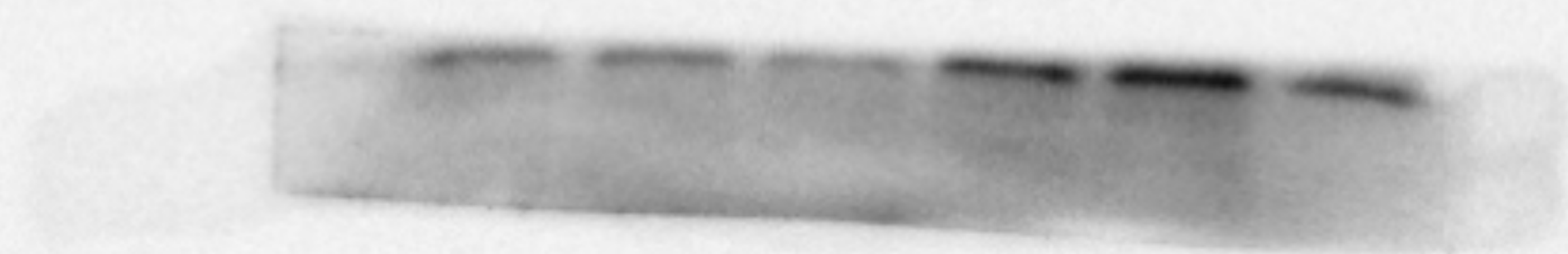

Supplement: Figure 6—source data 2. [file elife-86827-fig6-data2.zip › Figure 6-Source data 5 Raw unedited blots for Figure 6.pdf]

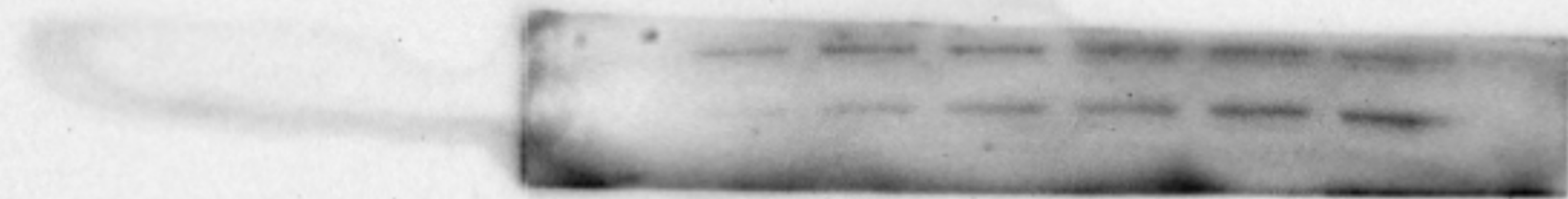

Supplement: Figure 6—source data 2. [file elife-86827-fig6-data2.zip › Figure 6-Source data 1 Raw unedited blots for Figure 6.pdf]

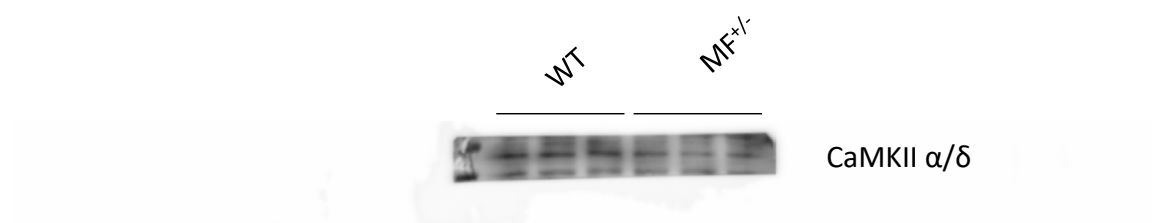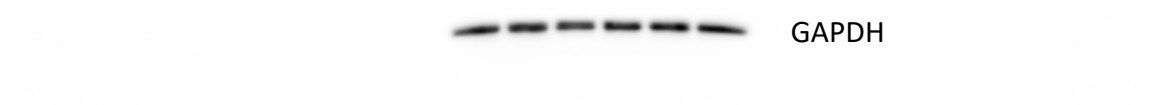

Supplement: Figure 8—source data 1. [file elife-86827-fig8-data1.zip › Figure 8-Source data 1 Uncropped and labeled blots for Figure 8.pdf]

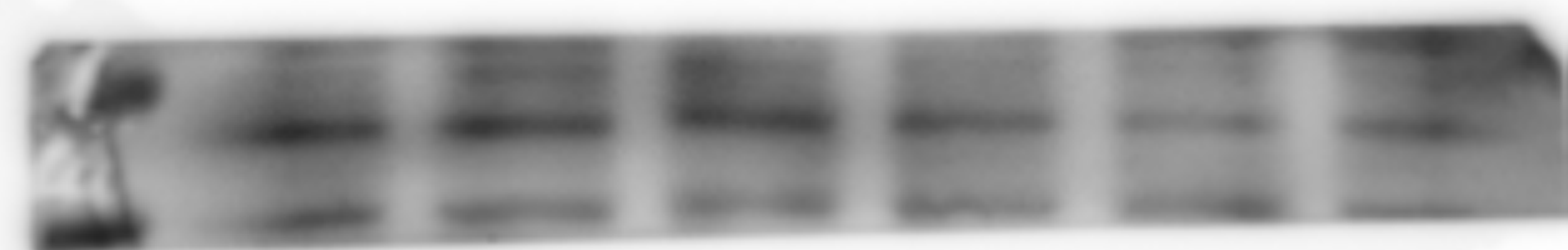

Supplement: Figure 8—source data 2. [file elife-86827-fig8-data2.zip › Figure 8-Source data 1 Raw unedited blots for Figure 8.pdf]
