# Supplementary material for: A MSTNDel73C mutation with FGF5 knockout sheep by CRISPR/Cas9 promotes skeletal muscle myofiber hyperplasia
Source: eLife. 2024 Oct 4;12:RP86827. doi: 10.7554/eLife.86827 (PMC11452178; doi:10.7554/eLife.86827)
Supplement: Supplementary file 1. — (A) Summary of generation of sheep carrying biallelic mutations in dual genes via the CRISPR/Cas9 system. (B) Muscle weight of different parts in WT and MF+/- sheep (g). (C) The slaughter traits of muscles in WT and MF+/- sheep. (D) Meat quality of longissimus dorsi in WT and MF+/- sheep. (E) Shearing force of different parts in WT and MF+/- sheep (N). (F) Amino acid content of longissimus dorsi in WT and MF+/- sheep (%, DM basis). [file elife-86827-supp1.docx]

## A *MSTN*^Del73C^ mutation with *FGF5* knockout sheep by CRISPR/Cas9 promotes skeletal muscle myofiber hyperplasia

Ming-Ming Chen^1,^ ^†^, Yue Zhao^1, †^, Kun Yu^1, †^, Xue-Ling Xu^1^, Xiao-Sheng Zhang^2^, Jin-Long Zhang^2^, Su-Jun Wu^1^, Zhi-Mei Liu^1^, Yi-Ming Yuan^1^, Xiao-Fei Guo^2^, Shi-Yu Qi^1^, Guang Yi^1^, Shu-Qi Wang^1^, Huang-Xiang Li^1^, Ao-Wu Wu^1^, Guo-Shi Liu^1^, Shoulong Deng^3^, Hong-Bing Han^1^, Feng-Hua Lv^1, *^, Di Lian^4, *^, Zheng-Xing Lian^1, *^

^1^ State Key Laboratory of Animal Biotech Breeding, Beijing Key Laboratory for Animal Genetic Improvement, National Engineering Laboratory for Animal Breeding, Key Laboratory of Animal Genetics and Breeding of the Ministry of Agriculture, College of Animal Science and Technology, China Agricultural University, Beijing 100193, China

^2^ Institute of Animal Husbandry and Veterinary Medicine, Tianjin Academy of Agricultural Sciences, Tianjin 300381, China

^3^ National Center of Technology Innovation for animal model, NHC Key Laboratory of Human Disease Comparative Medicine, Institute of Laboratory Animal Sciences, Chinese Academy of Medical Sciences and Comparative Medicine Center, Peking Union Medical College, Beijing, China

^4^ College of Pulmonary and Critical Care Medicine, Chinese PLA General Hospital, Beijing, China

† These authors contributed equally to this work.

* Correspondence:

[lianzhx@cau.edu.cn](mailto:lianzhx@cau.edu.cn) (Zheng-Xing Lian), [b20173020099@cau.edu.cn](mailto:b20173020099@cau.edu.cn) (Di Lian), [lvfenghua@cau.edu.cn](mailto:lvfenghua@cau.edu.cn) (Feng-Hua Lv)

### Supplementary file 1 Production and slaughter information of *MSTN*^Del73C^ mutation with *FGF5* knockout sheep

### Supplementary file 1A Summary of generation of sheep carrying biallelic mutations in dual genes via the CRISPR/Cas9 system

| Cas9/sgRNA  (molar ratio) | Embryo injected | Recipients | Pregnancy | Alive lambs | Mutation | | | Biallelic mutation | | |
| --- | --- | --- | --- | --- | --- | --- | --- | --- | --- | --- |
|  |  |  |  |  | MSTN | FGF5 | MSTN+FGF5 | MSTN | FGF5 | MSTN+FGF5 |
| 1:2 | 448 | 93 | 35 | 28 | 1 | 3 | 1 | 0 | 0 | 0 |
| 1:10 | 365 | 84 | 26 | 22 | 2 | 2 | 2 | 2 | 2 | 2 |
| 1:15 | 388 | 59 | 17 | 14 | 1 | 0 | 0 | 1 | 0 | 0 |

### Supplementary file 1B Muscle weight of different parts in WT and MF^+/-^ sheep (g)

| **Muscle Classification** | **WT (n=3)** | **MF^+/-^ (n=4)** | ***P*-value** |
| --- | --- | --- | --- |
| Longissimus dorsi | 905.67±37.15 | 760.00±48.68 | 0.077 |
| Biceps brachii | 50.67±2.67 | 48.25±3.2 | 0.606 |
| Triceps brachii | 247.33±18.41 | 265.00±8.01 | 0.374 |
| Gluteus medius | 305.00±25.24 | 339.75±20.1 | 0.324 |
| Semimembranous | 199.33±21.84 | 194.00±18.5 | 0.859 |
| Semitendinosus | 435.33±41.73 | 400.00±19.71 | 0.438 |
| Biceps femoris | 712.00±57.65 | 619.50±78.39 | 0.417 |
| Quadriceps femoris | 604.67±46.23 | 612.50±29.5 | 0.886 |

### Supplementary file 1C The slaughter traits of muscles in WT and MF^+/-^ sheep

| **Slaughter Indexes** | **WT (n=3)** | **MF^+/-^ (n=4)** | ***P*-value** |
| --- | --- | --- | --- |
| Live weight (kg) | 56.33±3.088 | 50.15±2.058 | 0.14201 |
| Carcass weight(kg) | 32.23±2.436 | 28.5±1.588 | 0.23588 |
| Slaughter percentage (%) | 57.12±1.237 | 56.75±1.259 | 0.84403 |
| loin muscle area (cm^2^) | 17.17±1.58 | 13.95±1.757 | 0.24795 |
| Meat weight (kg) | 18.79±1.306 | 15.68±0.825 | 0.08707 |
| The proportion of meat in carcass | 0.58±0.005 | 0.55±0.018 | 0.18691 |
| The proportion of brisket and neck meat | 0.14±0.018 | 0.13±0.004 | 0.85322 |
| The proportion of loin meat | 0.11±0.005 | 0.09±0.011 | 0.33339 |
| The proportion of rib meat | 0.22±0.004 | 0.15±0.003 | 0.00003 |
| The proportion of foreleg meat | 0.18±0.009 | 0.21±0.012 | 0.20974 |
| The proportion of hind leg meat | 0.33±0.009 | 0.4±0.016 | 0.0252 |
| Neat percentage (%) | 0.58±0.005 | 0.55±0.018 | 0.18691 |

### Supplementary file 1D Meat quality of longissimus dorsi in WT and MF^+/-^ sheep

| **Meat Quality** | **WT (n=3)** | **MF^+/-^ (n=4)** | ***P*-value** |
| --- | --- | --- | --- |
| pH_45min_ | 6.37±0.136 | 6.38±0.043 | 0.95934 |
| pH_24h_ | 5.58±0.038 | 5.49±0.038 | 0.230 |
| L* | 32.14±2.165 | 29.81±2.165 | 0.326 |
| a* | 13.44±0.452 | 15.29±0.452 | 0.084 |
| b* | 7.52±0.990 | 6.22±0.990 | 0.224 |
| Drip loss (%) | 24.84±1.802 | 26.11±1.802 | 0.446 |
| Cooking loss (%) | 30.62±0.864 | 29.15±0.864 | 0.314 |

### Supplementary file 1E Shearing force of different parts in WT and MF^+/-^ sheep (N)

| **Muscle Classification** | **WT (n=3)** | **MF^+/-^ (n=4)** | ***P*-value** |
| --- | --- | --- | --- |
| Longissimus dorsi | 42.7±5.497 | 40.5±6.080 | 0.808 |
| Biceps brachii | 37.7±2.125 | 40.2±8.675 | 0.806 |
| Triceps brachii | 49.8±7.174 | 55.5±5.023 | 0.530 |
| Gluteus medius | 53.2±4.946 | 68.0±7.206 | 0.181 |
| Semimembranous | 54.2±3.786 | 64.3±8.376 | 0.379 |
| Semitendinosus | 49.6±4.476 | 45.1±6.445 | 0.616 |
| Biceps femoris | 37.3±6.467 | 54.1±4.721 | 0.084 |
| Quadriceps femoris | 47.2±3139 | 57.0±7.298 | 0.325 |

### Supplementary file 1F Amino acid content of longissimus dorsi in WT and MF^+/-^ sheep (%, DM basis)

| **Amino Acid** | **WT (n=3)** | **MF^+/-^ (n=4)** | ***P*-value** |
| --- | --- | --- | --- |
| Aspartic acid (Asp) | 5.62±0.449 | 6.22±0.348 | 0.334 |
| Threonine (Thr) | 2.68±0.211 | 3.00±0.162 | 0.271 |
| Serine (Ser) | 2.11±0.164 | 2.40±0.133 | 0.222 |
| Glutamic acid (Glu) | 10.71±0.848 | 12.18±0.61 | 0.207 |
| Proline (Pro) | 2.04±0.164 | 2.33±0.134 | 0.224 |
| Glycine (Gly) | 2.62±0.203 | 2.92±0.171 | 0.311 |
| Alanine (Ala) | 3.47±0.272 | 3.87±0.210 | 0.285 |
| Cystine (Cys) | 0.54±0.050 | 0.57±0.029 | 0.707 |
| Valine (Val) | 3.20±0.256 | 3.52±0.196 | 0.356 |
| Methionine (Met) | 1.70±0.139 | 1.87±0.101 | 0.353 |
| Isoleucine (Ile) | 3.03±0.246 | 3.38±0.183 | 0.295 |
| Leucine (Leu) | 5.09±0.406 | 5.62±0.318 | 0.346 |
| Tyrosine (Tyr) | 2.11±0.186 | 2.06±0.338 | 0.912 |
| Phenylalanine (Phe) | 3.15±0.279 | 3.48±0.207 | 0.376 |
| Lysine (Lys) | 5.32±0.437 | 5.97±0.358 | 0.297 |
| Histidine (His) | 2.22±0.200 | 2.38±0.179 | 0.577 |
