# Supplementary material for: A MSTNDel73C mutation with FGF5 knockout sheep by CRISPR/Cas9 promotes skeletal muscle myofiber hyperplasia
Source: eLife. 2024 Oct 4;12:RP86827. doi: 10.7554/eLife.86827 (PMC11452178; doi:10.7554/eLife.86827)
Supplement: Supplementary file 2. — (A) Primers sequences of gene cloning. (B) The sequences of siRNA. (C) All primers of PCR and RT-qPCR. (D) The antibodies information. [file elife-86827-supp2.docx]

## A *MSTN*^Del73C^ mutation with *FGF5* knockout sheep by CRISPR/Cas9 promotes skeletal muscle myofiber hyperplasia

Ming-Ming Chen^1,^ ^†^, Yue Zhao^1, †^, Kun Yu^1, †^, Xue-Ling Xu^1^, Xiao-Sheng Zhang^2^, Jin-Long Zhang^2^, Su-Jun Wu^1^, Zhi-Mei Liu^1^, Yi-Ming Yuan^1^, Xiao-Fei Guo^2^, Shi-Yu Qi^1^, Guang Yi^1^, Shu-Qi Wang^1^, Huang-Xiang Li^1^, Ao-Wu Wu^1^, Guo-Shi Liu^1^, Shoulong Deng^3^, Hong-Bing Han^1^, Feng-Hua Lv^1, *^, Di Lian^4, *^, Zheng-Xing Lian^1, *^

^1^ State Key Laboratory of Animal Biotech Breeding, Beijing Key Laboratory for Animal Genetic Improvement, National Engineering Laboratory for Animal Breeding, Key Laboratory of Animal Genetics and Breeding of the Ministry of Agriculture, College of Animal Science and Technology, China Agricultural University, Beijing 100193, China

^2^ Institute of Animal Husbandry and Veterinary Medicine, Tianjin Academy of Agricultural Sciences, Tianjin 300381, China

^3^ National Center of Technology Innovation for animal model, NHC Key Laboratory of Human Disease Comparative Medicine, Institute of Laboratory Animal Sciences, Chinese Academy of Medical Sciences and Comparative Medicine Center, Peking Union Medical College, Beijing, China

^4^ College of Pulmonary and Critical Care Medicine, Chinese PLA General Hospital, Beijing, China

† These authors contributed equally to this work.

* Correspondence:

[lianzhx@cau.edu.cn](mailto:lianzhx@cau.edu.cn) (Zheng-Xing Lian), [b20173020099@cau.edu.cn](mailto:b20173020099@cau.edu.cn) (Di Lian), [lvfenghua@cau.edu.cn](mailto:lvfenghua@cau.edu.cn) (Feng-Hua Lv)

### Supplementary file 2 The information of primers sequence, siRNA sequence and antibody

### Supplementary file 2A Primers sequences of gene cloning

| Genes | Primer Name | Primer Sequences（5’-3’） |
| --- | --- | --- |
| FOSL1 | pFOSL1-F | tttgccgccagaacacaggaccggttctagaGCCACC**atgttccgagactacggggaac** |
|  | pFOSL1-R | agagagaagtttgttgcgccggatcccttatcgtcatcgtctttgtaatc**taaggccaagagggttggggag** |

Note: The bold is the FOSL1 gene sequences, the Kozak sequence in the box, the underline position is the flag tag sequence, and the lowercase letters are the homologous arm sequence of the vector.

### Supplementary file 2B The sequences of siRNA

| Sequences Name | Sequences (5’-3’) |
| --- | --- |
| si-oar-FOSL1_001 | GGAAAGAACTGACCGACTT |
| si-oar-FOSL1_002 | AACCCTCTTGGCCTTATGA |
| si-oar-FOSL1_003 | CCAAGCATCAACACTGTGA |

### Supplementary file 2C All primers of PCR and RT-qPCR

| Genes |  | Primer sequences（5’-3’） | Product length (bp) |
| --- | --- | --- | --- |
| MSTN | F  R | AAGTCAAGGTAACAGACACACC  CAATACTACATATAGATTTTTC | 756 |
| FGF5 | F  R | ATTTCAAGAAAACAGCTATAAT  AAAAGTTGCCTTCAGAGCACT | 396 |
| GAPDH | F | GTCGGAGTGAACGGATTTGG | 97 |
|  | R | TGAAGGGGTCATTGATGGCA |  |
| MSTNdel | F | CAATTACTGCTCTGGAGAATGTG | 121 |
|  | R | AGACATCTTTGTAGGAGTACAGC |  |
| Cyclin A1 | F | CCAAGGCACACTACATGAGGAA | 133 |
|  | R | AGGAAGTTGACAGCCAGGTAGA |  |
| Cyclin B1 | F | TGACTGACAACACCTACACCAA | 142 |
|  | R | AGCTCAACATCAACCTCTCCAA |  |
| Cyclin D1 | F | CATCGAGCACTTCCTCTCCAA | 111 |
|  | R | GAAATGAACTTCACGTCTGTGGC |  |
| Cyclin E1 | F | CAGTGTGGCAGTCAGCCTTG | 123 |
|  | R | CCTCTGAGGCTTGTACGCAG |  |
| CDK1 | F | CATGAGGTAGTGACACTCTGGTA | 127 |
|  | R | ACAGTGGTTTCTTCGTTGCTAA |  |
| CDK2 | F | TACCCCATACCCCGTGACCC | 108 |
|  | R | GGCCAAACCACCTCATCTGG |  |
| CDK4 | F | ACATTCTGGTGACAAGTGGTGG | 85 |
|  | R | GGTGTAAGTGCCATCTGGTAGC |  |
| CDK6 | F | GCCTGGACTTTCTTCATTCTCAC | 144 |
|  | R | CCACTGAGGTAAGAGCCATCTG |  |
| PCNA | F | CAAGTGGCGTGAACCTACAG | 142 |
|  | R | AGTATTTTGGACATGCTGGTGAG |  |
| BCL2 | F | TTGGGAAGTTTTCAGAGCAGC | 140 |
|  | R | CCTCCTCCGTGATGTGGTAT |  |
| MKI67 | F | CTGGTGTCAAGAGTCGGCTAAGA | 141 |
|  | R | GGCAGGACGCTGGAGTGATT |  |
| MyoD1 | F | CCCCAACCCGATTTACCAGG | 119 |
|  | R | TAAGCGCAATCTTTTGGGCG |  |
| MyoG | F | CCAGTGAATGCAGCTCCCATA | 132 |
|  | R | AGGTGAGGGAGTGCAGATTG |  |
| MyHC | F | ATGAGGGGGACACTGGAAGA | 113 |
|  | R | CGGATGAACTTGCCAAAGCG |  |
| FOSL1 | F | GGTGTTTCTGATGCTCGCTG | 113 |
|  | R | GGCTAGAGCTTGATGCGGTT |  |
| c-Fos | F | AAGGAGAATCCGAAGGGAAAGG | 100 |
|  | R | TAGTTGGTCTGTCTCCGCTT |  |
| cMyoD1-1 | F | GACGGACTGCAAGGAGGAAG | 147 |
|  | R | GCTCAGATCGCTCCAATATCCA |  |
| cMyoD1-2 | F | CATCGTCACAGGTGGCATCA | 90 |
|  | R | TCTCCAGCACCCTGACTAAATC |  |
| Rac1 | F | ACCCGCAGACAGATGTATTCT | 139 |
|  | R | TCAAGTTTCGTCCCCACCAG |  |
| RYR1 | F | CCACAACTTTAAGCGCGAGG | 113 |
|  | R | ACTGTACATCTCCCGCCTTG |  |
| RYR3 | F | AAAGTATGGGCCCGAAGTGG | 107 |
|  | R | CCGCCTGTGCTTTCCTTTTC |  |
| MYMK | F | CCAGAAGGCGGTTCCACAT | 146 |
|  | R | CGTCCCGTAGATGCTGAAGTA |  |
| MYMX | F | GCTGTCTGTTGTTCGTCCTCA | 90 |
|  | R | CTAGCCTCTCCCTCCTTTCCA |  |
| DMD | F | TGCACTACTCTCAACAGCGT | 131 |
|  | R | GGCTGAAAGGAGCCATGAGA |  |
| SMN1 | F | GGTGTAGCTCTCTCCCAAGGA | 109 |
|  | R | ATGCAGATGACATGCACCCA |  |
| MTM1 | F | CCAGTGGAGCAGCGTTACAT | 110 |
|  | R | GAGGCCGAGGGATCAGAAAG |  |
| IGF1 | F | TCACATCCTCCTCGCATCTCTT | 130 |
|  | R | CTGTCTCCGCACACGAACTG |  |
| GAA | F | CAGCCGCAGGAACCATACAG | 107 |
|  | R | CCGTGGAACAGCGTGTAGAG |  |
| ACVR1 | F | TGCCTTCTAGCCTGCCTACTG | 117 |
|  | R | GTTGGTGGTGATGAGCCCTTC |  |
| ACVR2A | F | CCAGTAACACCTAAGCCTCCC | 142 |
|  | R | GAGTTGGAACGAGCACAGGA |  |
| ACVR2B | F | CCAGCTCTGCGTGACAATC | 107 |
|  | R | CGTTGACCGACCTCCGAAT |  |

### Supplementary file 2D The antibodies information

| Antibody Name | Manufacturer | Catalog Number | Host |
| --- | --- | --- | --- |
| GAPDH | Zhongshan Golden Bridge | TA-08 | Mouse |
| β-Tubulin | Zhongshan Golden Bridge | TA-10 | Mouse |
| MyoD1 | Affinity | AF7733 | Rabbit |
| MyoG | DSHB | F5D | Mouse |
| MyHC | DSHB | MF20 | Mouse |
| FOSL1 | Affinity | AF6921 | Rabbit |
| p-FOSL1 | Affinity | AF7235 | Rabbit |
| MEK1/2 | Affinity | AF6385 | Rabbit |
| p-MEK1 | Beyotime | AF1786 | Rabbit |
| ERK1/2 | Affinity | 4695 | Rabbit |
| p-ERK1/2 | Affinity | 4370 | Rabbit |
| p38 MAPK | Affinity | AF6456 | Rabbit |
| p-p38 MAPK | Affinity | AF4001 | Rabbit |
| Pax7 | DSHB | - | Mouse |
| Myosin VIIa | abcam | Ab3481 | Rabbit |
| MSTN | Affinity | DF13273 | Rabbit |
